# Selective acquired long QT syndrome (saLQTS) upon risperidone treatment

**DOI:** 10.1186/1471-244X-12-220

**Published:** 2012-12-05

**Authors:** Maciej Jakub Lazarczyk, Zahir A Bhuiyan, Nicolas Perrin, Panteleimon Giannakopoulos

**Affiliations:** 1 Division of General Psychiatry, University Hospitals of Geneva and Faculty of Medicine of the University of Geneva, 1202 Geneva, Switzerland; 2 Division of Old Age Psychiatry, Hospices-CHUV, 1008, Prilly, Switzerland; 3 Laboratoire de Génétique Moléculaire, Service de Génétique Médicale, Centre Hospitalier Universitaire Vaudois, Lausanne, Switzerland

**Keywords:** Long QT syndrome, Acquired long QT syndrome, Selective acquired long QT syndrome, QT, Antipsychotic, Risperidone, Clozapine, KCNH2, hERG

## Abstract

**Background:**

Numerous structurally unrelated drugs, including antipsychotics, can prolong QT interval and trigger the acquired long QT syndrome (aLQTS). All of them are thought to act at the level of KCNH2, a subunit of the potassium channel. Although the QT-prolonging drugs are proscribed in the subjects with aLQTS, the individual response to diverse QT-prolonging drugs may vary substantially.

**Case presentation:**

We report here a case of aLQTS in response to small doses of risperidone that was confirmed at three independent drug challenges in the absence of other QT-prolonging drugs. On the other hand, the patient did not respond with QT prolongation to some other antipsychotics. In particular, the administration of clozapine, known to be associated with higher QT-prolongation risk than risperidone, had no effect on QT-length. A detailed genetic analysis revealed no mutations or polymorphisms in *KCNH2*, *KCNE1*, *KCNE2, SCN5A* and *KCNQ1* genes.

**Conclusions:**

Our observation suggests that some patients may display a selective aLQTS to a single antipsychotic, without a potassium channel-related genetic substrate. Contrasting with the idea of a common target of the aLQTS-triggerring drugs, our data suggests existence of an alternative target protein, which unlike the KCNH2 would be drug-selective.

## Background

The first cases of reversible QT prolongation upon exposure to environmental stressors (mostly drugs) have been described in the 1970s and designated as the “acquired long QT syndrome” (aLQTS). The aLQTS-triggering drugs are supposed to act at the subunit of the potassium channel, the KCNH2, which produces the delayed rectifier K(+) current (I(Kr)) [1]. The KCNH2 is blocked by numerous structurally unrelated drugs (antipsychotics and antiarrhythmics) that affect cardiac repolarization [2-4]. Although the effect of this blockage is clinically irrelevant in general population, in a small proportion of subjects, otherwise asymptomatic, the presence of mutations in the *KCNH2* or other functionally related genes potentiates the effect of these drugs resulting in a massive QT-prolongation and an overt aLQTS [5-7]. Given the serious health repercussions of this phenomenon, the aLQTS-triggering drugs are all proscribed in these vulnerable individuals, since they are supposed to have a common mechanism of QT-prolongation.

However, we describe here a patient, who in the absence of genetic alterations in the LQTS-related genes, selectively responds with QT prolongation to risperidone only. This observation suggests the presence of an alternative target protein that unlike the KCNH2 would be drug-selective.

## Case report

**Figure 1:**
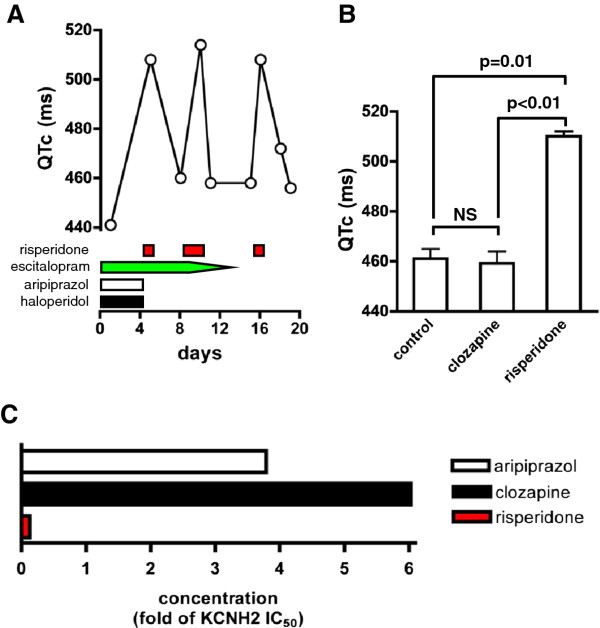
**A. Risperidone induces QT prolongation.** ECG at the admission was normal and the corrected QT value (QTc; according to the Bazett formula) was not prolonged under the treatment of aripiprazol (20 mg/day), haloperidol (3 mg/day) and escitalopram (20 mg/day). Subsequently, the QTc value was monitored regularly. At day 4, 9 and 15 risperidone (2 x 1 mg/day) was introduced and thereafter rapidly stopped at day 4, 10 or 15, respectively, as indicated on the graph. Other antipsychotics/antidepressant were discontinued, as indicated on the graph. QTc prolongation was observed each time when risperidone was introduced, even after a single 1 mg dose, and it returned to the normal range immediately after the risperidone treatment was stopped. **B. Comparative analysis of the impact of risperidone and clozapine on QT length.** QTc values have been measured during the treatment with progressively increasing doses of clozapine (12,5 – 275 mg; n=14), risperidone (1 – 2 mg; n=3) or in the absence of these drugs (control; n=8). Risperidone but not clozapine caused statistically significant QTc prolongation in this patient, as assessed by Mann–Whitney *U* test. NS – statistically not significant. **C. Plasma concentration of aripiprazol, clozapine and risperidone in relation to KCNH2 IC**_50_. Drug plasma concentration has been determined at the steady state, for aripiprazol and clozapine, or the second day after its introduction, for risperidone (2 x 1 mg/day), at the time of the predicted peak of the plasma drug level. On the graph, the drug concentrations have been expressed as a fold of their respective KCNH2 IC_50_values (263 nM for aripiprazol, 320 nM for clozapine, and 148 nM for risperidone), previously determined for these drugs. At the indicated concentrations, risperidone, but not aripiprazol or clozapine, significantly prolonged QT length.

We report here a case of 37-year old woman with schizophrenia, hospitalized for an exacerbation of psychotic symptoms. She had no personal/family history of cardiac diseases or sudden deaths. Besides benzodiazepines, she was treated at the admission with aripiprazol (20 mg/day), haloperidol (3 mg/day) and escitalopram (20 mg/day). The routine laboratory and clinical check-up (including ECG and blood electrolytes) revealed no abnormalities. The patient developed the aLQTS in response to small doses of risperidone (1–2 mg/day), confirmed at three independent drug challenges. Noteworthy, the patient responded with significant QT prolongation to risperidone (QTc increase from 458 to 508 ms), also when all other drugs, which might potentially affect QT length, were discontinued (Figure 1A). The reason of this extreme sensitivity to risperidone was unclear but the contribution of a cytochrome polymorphism or other elimination failures is unlikely since risperidone prolonged QT at very low blood concentrations (19.1 nM). Moreover, the concentration of paliperidone, an active metabolite of risperidone with QT-prolonging potential [8], was very low too, and the cumulative blood concentration of risperidone and paliperidone was subtherapeutic. Interestingly, the patient did not respond with QT prolongation following the administration of other antipsychotics (e.g. aripiprazol, clothiapine, haloperidol; data not depicted). In particular, the administration of clozapine, known to be associated with higher QT-prolongation risk than risperidone [9], had no effect on QT-length (Figure 1B).

We hypothesized that our patient carries a mutation or polymorphism in the *KCNH2*, which could determine conformational alterations of the channel and thus differentially affect its capacity to bind risperidone and clozapine. Therefore, we have sequenced all the coding exons and exon-intron boundaries of the *KCNH2* (isoforms 1a and 1b), but no mutation or polymorphism was found (data not depicted). We have also excluded any mutation in the *SCN5A* gene, as well as in the *KCNE2* (data not depicted), the gene encoding the β–subunit of the Ikr channel, as well as G38S and D85N polymorphism in the *KCNE1*, reported to cause the LQTS [10,11]. Since a subtle mutation in the *KCNQ1* could affect the repolarisation reserve, indirectly leading to the aLQTS [12,13], we have sequenced also this gene but found no mutation (data not depicted). Additionally, we have excluded any exonic deletion and duplication of the *KCNH2* by Multiplex Ligation-dependent Probe Amplification (data not depicted; P114-A2-LQT probemix, MRC-Holland).

## Conclusions

The QT prolongation in our patient fulfills the criteria for aLQTS. However, because of its striking drug-selectivity, we use the term “selective acquired long QT syndrome” (saLQTS). The present report indicates that some patients with drug-induced LQTS display in fact the saLQTS and would tolerate perfectly well certain KCNH2 blockers. Even though the saLQTS has not previously been reported, we suppose that its prevalence among the patients displaying drug-induced long QT could be underestimated since the response to diverse QT-prolonging drugs within the same subjects is only rarely investigated.

The molecular mechanism of the saLQTS in the reported case remains challenging. Since risperidone scarcely prolongs QTc (< 4 ms) in a general population (with *a priori* a wild-type *KCNH2*) [14], the sensitivity to this drug in our patient (also carrying only wild-type alleles of this gene) is most probably not mediated by the KCNH2. Despite having sequenced the gene we still cannot formally exclude a defect affecting the expression, trafficking, and cellular localization of the protein. However, it is highly unlikely that such a defect would selectively affect its interaction only with risperidone. It is also noteworthy that risperidone prolonged QT already at concentration much lower than its KCNH2 IC_50_ (half maximal inhibitory concentration), whereas other antipsychotics had no impact on QT in this patient, even at concentrations up to 6-times higher than their KCNH2 IC_50_ values (Figure 1C).

Sequencing of some other genes implicated in the pathogenesis of LQTS did not reveal any significant mutation or polymorphism in our patient, and exact molecular mechanism of the reported saLQTS remains unknown. Admittedly, it cannot be formally excluded that further expanding of genetic screening on all of the genes implicated in the LQTS could lead to the identification of causative mutations. However, this seems rather unlikely, since the mutations in the five most important genes that we have already sequenced (*KCNH2*, *KCNQ1*, *KCNE1*, *KCNE2* and *SCN5A*) are responsible for probably more than 95% of the cases of LQTS with known genetic background [15].

Taken together, these data imply the presence of a KCNH2-independent pathway leading to aLQTS. Although mutations in several genes have already been reported in this condition [7], it has been long considered that the corresponding proteins do not constitute a drug target but they rather modulate an outcome of the drug-KCNH2 interactions. Contrasting with this idea, our data suggest the existence of an alternative target protein, which unlike the KCNH2 would be drug-selective.

## Consent

Written informed consent was obtained from the patient for publication of this case report and any accompanying images. A copy of the written consent is available for review by the Series Editor of this journal.

## Abbreviations

aLQT syndrome: Acquired long QT syndrome; saLQT syndrome: Selective acquired long QT syndrome; IC_50_: Half maximal inhibitory concentration.

## Competing interests

The authors declare that they have no competing interests.

## Authors’ contributions

ML and NP treated and followed up the patient. ML and PG did literature survey, conceptualized and wrote the case report. NP helped in writing the manuscript. ML prepared the figure. ZB performed the genetic analysis and helped in writing the manuscript. All authors read and approved the final document.

## Pre-publication history

The pre-publication history for this paper can be accessed here:

http://www.biomedcentral.com/1471-244X/12/220/prepub
